# Utilization of growth monitoring and promotion services among children younger than 2 years in West Armachiho district, Northwest Ethiopia

**DOI:** 10.3389/fpubh.2023.1179720

**Published:** 2023-11-22

**Authors:** Novel Teklemuz, Mekonnen Sisay, Lemlem Daniel Baffa, Berhanu Mengistu, Azeb Atenafu

**Affiliations:** Department of Human Nutrition, Institute of Public Health, College of Medicine and Health Sciences, University of Gondar, Gondar, Ethiopia

**Keywords:** utilization, growth monitoring and promotion services, children younger than 2 years, West Armachiho, Northwest Ethiopia

## Abstract

**Introduction:**

Inadequate physical growth and poor development of children are prevalent and significant problems worldwide, with 149 million children younger than 5 years stunted and 49 million wasted. Growth monitoring and promotion (GMP) is one of the major activities implemented with the aim of capturing growth faltering before the child reaches the status of undernutrition. In relation to this, the Amhara region, where the study area is found, is a highly burdened area for child malnutrition. Thus, it needs further investigation about the utilization of GMP services and associated factors among children younger than 2 years in the study area.

**Objective:**

The aim of this study was to assess the utilization of growth monitoring and promotion services and associated factors among children younger than 2 years.

**Methods:**

A community-based cross-sectional study was conducted in the West Armachiho district, including 703 mother–child pairs, with a response rate of 94.7%. A simple random sampling technique was used to select the respondents. Both bivariable and multivariable logistic regression analyzes were performed. An adjusted odds ratio (AOR) with a 95% confidence interval was used to measure the strength of the association.

**Results:**

The proportion of utilization of growth monitoring and promotion services in the West Armachiho district was 13.7% (95%Cl; 11.2, 16.4). Factors such as maternal educational status (AOR = 2.17, 95%Cl; 1.05, 4.49), institutional delivery (AOR = 3.16, 95%Cl; 1.62, 6.13), family size (AOR = 2.66, 95%Cl; 1.13, 6.23), access to health facility (AOR = 3.17, 95%Cl; 1.45, 6.95), and maternal knowledge (AOR = 4.53, 95%Cl; 2.71, 7.59) were significantly associated with the utilization of growth monitoring and promotion services.

**Conclusion:**

Utilization of growth monitoring and promotion services in children younger than 2 years in the West Armachiho district was low. Thus, giving due attention to the improvement of the knowledge of the mothers/caregivers about child GMP services and counseling them about the importance of facility delivery is vital to improving growth monitoring and promotion services in the area.

## Introduction

Inadequate physical growth and poor development of children are prevalent and significant problems worldwide, making 149 million children younger than 5 years stunted and 45 million wasted (1), while their burden is very significant in the African region, where approximately 33.1% were stunted, 17.1% were underweight, and 7% were wasted in 2018 (2). This burden is significant among young Ethiopian children, where 27.21, 7.8, and 16.44% of children younger than 2 years are stunted, wasted, and underweight, respectively (3). According to the 2019 Ethiopian Mini-Demographic and Health Survey report, the Amhara region is the second highest region in terms of the prevalence of stunting, where 42% of children younger than 5 years are stunted, showing that child growth and development is a huge problem in this area.

GMP is a nutrition-related preventive activity for young children alongside Oral rehydration, Breastfeeding, and Immunization (GOBI) (4), which is applied by measuring and interpreting growth to facilitate communication and interaction with caregivers and generate adequate action to promote child growth. It is used as a screening tool, for education and promotional purposes, for nutrition surveillance, and as an integrating strategy (5–8).

Growth monitoring is an important component of all child health services. It is part of the United Nations International Children’s Emergency Fund’s (UNICEF) or World Health Organization’s (WHO) child survival strategy and the goal set at the World Summit on Children (9). GM should start at birth and continue until the child is 23 months of age, and it is carried out at the health facility and/or community levels (10). As weight gain is believed to be the most sensitive indicator of growth, the most widely promoted method of growth monitoring is weight gain and charting growth (11). In Ethiopia, the utilization of GMP by children younger than 2 years ranges from 16.9% in southern Ethiopia to 38.9% in northeastern Ethiopia (12–15), which is affected by several factors such as the educational status of the mother, the age of the index child, family size, place of delivery, household wealth status, availability and accessibility of health facilities, physical presence and distance from home to health facilities, maternal knowledge and attitude toward GMP, counseling on GMP, and having ANC and PNC services (12, 13, 16, 17).

Currently, the government of Ethiopia is working on prevention and promotion activities to fight malnutrition in children by designing several nutrition-specific and sensitive interventions. One of these is GMP, which is implemented under the health extension program packages to ensure that younger children are not missed during the critical window of opportunity to break the intergenerational cycle of malnutrition and capture growth faltering before the child reaches the status of undernutrition (18).

However, the lack of awareness among mothers about childhood malnutrition and the GMP program was the major challenge, according to a qualitative study conducted in Ethiopia (19), which exacerbates the burden of undernutrition in the country. In relation to this, the Amhara region, where the study area is found, is a highly burdened area for child malnutrition, especially stunting (20). In addition to this, there are very few studies conducted in Ethiopia to assess the service utilization by the mothers or the caregivers, and no study is mentioned to show the real image of utilization of GMP services in the study area, making the situation difficult for the early design and application of other appropriate nutrition interventions. Therefore, this study will determine the magnitude of the utilization of growth monitoring and promotion (GMP) services and factors affecting it among children younger than 2 years in West Armachiho district, Ethiopia.

## Materials and methods

### Study design and setting

A community-based cross-sectional study was conducted from February 2020 to March 2020 in West Armachiho district, one of the six districts in the West Gondar zone located in the Amhara Regional State, Ethiopia. The district is located 944 km northwest of Addis Ababa, the capital of Ethiopia. The district consists of 17 kebeles (the smallest administrative units in Ethiopia) with an estimated 2019 annual projection, a population of 49,296, and approximately 2,489 children younger than 2 years old. There is 1 primary hospital, 3 health centers, and 11 health posts in the district. Maize, sorghum, teff, legumes, potato, spinach, lettuce, cabbage, kale, pumpkin, mango, avocado, banana, and orange are the main food products produced in the area.

### Population, sample size determination, sampling techniques, and procedures

All mothers with children aged 0 to 23 months who lived for at least 6 months in randomly selected kebeles were included in the study. Mothers with children aged 0 to 23 months who were seriously ill and unable to communicate were excluded from the study. The sample size was determined using a single population proportion formula by considering the assumption of a 16.9% proportion of utilization of GMP services obtained from a study conducted in Mareka district (13), 95% confidence interval, 4% margin of error, 10% non-response rate, and a design effect of 2, which yielded 742 mother–child pairs.

Of the 17 kebeles in the district, 6 (2 urban and 4 rural) kebeles were selected by simple random sampling. Then, households having mothers with children aged 0–23 months in each selected kebele were selected by simple random sampling. Proportional allocation was used to select representative samples from each kebele.

### Operational definitions and study variables

#### Utilization of GMP services

Participation of a child in GMP services at least once for 0 months, at least two times for 1–3 months, at least five times for 4–11 months, and at least four times per year for 12–23 months (21).

#### Positive attitude

Those mothers/caregivers who score 3 or higher on 5 attitude questions (16).

#### Good knowledge

Those who score 8 or higher on the 18 knowledge questions (16).

#### Wealth quintile

The wealth quintile was assessed by principal component analysis (PCA) from variables containing agricultural productivity, fixed assets, household assets, and utility, which are categorized as lowest, second, middle, fourth, and highest (20).

### Data collection tool, procedure, and quality control

The data were collected using a structured questionnaire via face-to-face interviews. The questionnaire was designed to capture the sociodemographic and economic characteristics of the respondents, the availability and accessibility of health facilities and health services, and maternal knowledge and attitude toward child growth monitoring and promotion. It was originally prepared in English and translated to Amharic (the local language of the respondents) and back to English to ensure consistency. Two supervisors and four data collectors participated in the data collection process. Both data collectors and supervisors were trained on data quality, informed consent, and interview techniques. The data collection tool was pretested on 5% of the total sample of similar respondents and set outside the actual study setting (Teda area). During the pretest, some limitations, such as the use of vague words and the inclusion of a wealth index measuring crops that do not grow in the study area, such as coffee, wheat, and beans, were noticed in the questionnaire. Following this, appropriate modifications, such as replacing the vague words and removing the inappropriately included crops, were performed after the completion of the pretest. The principal investigator daily monitored the data collection process, and the supervisors spot-checked and reviewed the completed copies of the questionnaires for completeness and consistency.

### Data processing and analysis

After the data collection, each questionnaire was checked for completeness and cleaned. Then, it was entered into Epi info version 7.2 and then transferred to SPSS version 20 for analysis. Descriptive statistics were performed for the description of the characteristics of the respondents and presented using texts, tables, and graphs. A chi-square test was performed for all variables to check the assumptions. A binary logistic regression model was fitted to identify factors associated with the utilization of GMP services. Accordingly, variables with a value of p of <0.2 in the bivariate analysis were considered for multivariable logistic regression analysis. Variables with a value of p of <0.05% were considered statistically significant in the multivariate analysis. Both crude and adjusted odds ratios with the corresponding 95% confidence interval were calculated to measure the strength and presence of an association between the utilization of GMP services and its determinants. A Hosmer–Lemeshow goodness of fit test was run to test the model’s fitness, and it showed that the model was adequate (*p*-value = 0.76). Furthermore, multicollinearity was not found between independent variables. The wealth index of the respondents was analyzed using PCA.

## Results

### Sociodemographic and economic characteristics

A total of 703 mothers with children aged 0–23 months were interviewed, for a response rate of 94.7%. The mean (±SD) age of mothers/caregivers was 29.8 ± 5.97 years. The majority (62.3%) of mothers/caregivers did not have formal education, while 56.3% of them were housewives. Approximately half (52.2%) of the children were younger than 11 months. A little more than half of the study subjects (51.4%) had a household family size of 4–5 (Table 1).

**Table 1:** Sociodemographic and economic characteristics of the respondents in West Armachiho district, Ethiopia 2020.

Variables	Category	Frequency	Percentage (%)
Sex	Male	342	48.6
	Female	361	51.4
Child’s age	0–11	366	52.1
	12–17	187	26.6
	18–23	150	21.3
Maternal age	15–24	128	18.2
	25–34	429	61.0
	35–44	123	17.5
	> = 45	23	3.3
Maternal ethnicity	Amhara	656	93.3
	Tigray	39	5.5
	Other	8	1.1
Maternal education	No formal education	438	62.3
	Primary	201	28.6
	Secondary and above	64	9.1
Maternal employment	Housewife	396	56.3
	Farmer	210	29.9
	Merchant	50	7.1
	Government employee	30	4.3
	Others	17	2.4
Husband education	No formal education	375	53.3
	Primary	221	31.4
	Secondary and above	107	15.2
Residence	Urban	394	56
	Rural	309	44
Family size	<4	135	19.2
	4–5	361	51.4
	>5	207	29.4
Wealth index	Lowest	106	15.1
	Second	171	24.3
	Middle	135	19.2
	Fourth	141	20.1
	Highest	150	21.3

### Mothers/caregivers’ health service utilization

From the total respondents, more than two-thirds (68.8%) of mothers or caregivers had ANC follow-up, while 53.9% of the respondents gave birth at home. Approximately 70% of the respondents have access to health facilities within 5 km or fewer.

### Knowledge and attitude on the utilization of GMP services

Among the total respondents, only 19.3% had good knowledge and 8.8% had a positive attitude toward growth monitoring and promotion services.

### Utilization of growth monitoring and promotion services

**Figure 1:**
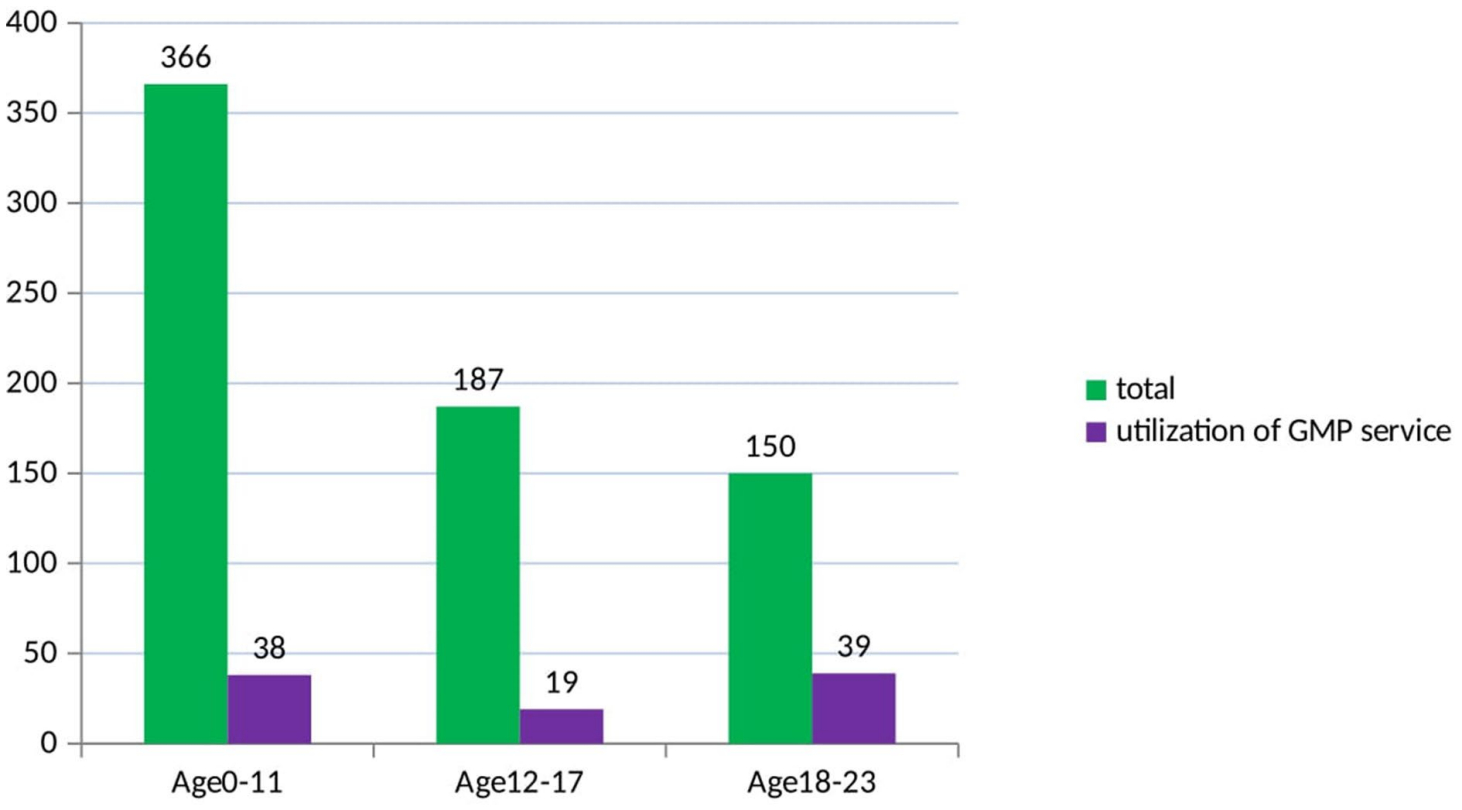
Utilization of growth monitoring and promotion service based on age category of children of age less than two years in West Armachiho district, Northwest ethiopia.

The result of this study showed that the utilization of growth monitoring and promotion services among children younger than 2 years in West Armachiho was 13.7% (95%Cl; 11.2, 16.4; Figure 1).

### Factors associated with the utilization of growth monitoring and promotion services

The association between the utilization of GMP services and independent variables was analyzed using a binary logistic regression model. All variables that fulfilled the chi-square assumptions with a value of p of *<*0.2 in bivariable analysis were fitted into the multivariable logistic regression model. Hence, delivery place, family size, husband’s educational status, accessibility of health facilities, wealth status, good maternal knowledge, ANC follow-up, and maternal education were included in the multivariable logistic regression analysis. Accordingly, delivery place, family size, accessibility of health facilities, good maternal knowledge, and maternal education were found to be statistically significant at a value of p of <0.05 in the multivariable analysis and were considered to be associated with the utilization of growth monitoring and promotion services.

The odds of using GMP services were 3.2 (AOR = 3.16, 95%Cl; 1.629, 6.136) times higher among mothers/caregivers who delivered at the health facility compared to those who delivered at home. Similarly, mothers with less than four family members were 2.7 (AOR = 2.661, 95%Cl; 1.135, 6.239) times more likely to use GMP services than their counterparts. Similarly, the odds of the utilization of GMP services were 3.17 (AOR = 3.174, 95%Cl; 1.450, 6.952) times higher among mothers/caregivers who have access to a health facility compared to those who do not. In addition, mothers/caregivers who had good knowledge about GMP services were 4.53 (AOR = 4.537, 95%CI; 2.711, 7.594) times more likely to use the services as compared to their counterparts. Furthermore, the likelihood of using GMP services was 2.17 (AOR = 2.174, Cl95%; 1.051, 4.498) times higher among mothers/caregivers who had attended primary school compared to those mothers who did not have formal education (Table 2).

**Table 2:** Factors associated with the utilization of growth monitoring and promotion services among mothers with children younger than 2 years in West Armachiho district, northwest Ethiopia 2020.

Variable		Utilization of GMP services		95%Cl		*P*-value
		Yes	No	COR	AOR	
Delivery place	Home	18	361	1	1	
	Health institution	78	246	6.35 (3.71,10.88)	3.16 (1.62,6.13)	0.001*
Family size	<4	30	105	5.09 (2.45,10.56)	2.66 (1.13,6.23)	0.024*
	4–5	55	306	3.20 (1.63,6.27)	2.00 (0.93,4.30)	0.074
	>5	11	196	1	1	
Husband education	No formal education	18	357	1	1	
	Primary	47	174	5.35 (3.02,9.49)	1.57 (0.71,3.43)	0.258
	Secondary and above	31	76	8.09 (4.30,15.21)	1.24 (0.46,3.37)	0.663
Knowledge	Poor	47	520	1	1	
	Good	49	87	6.23 (3.93,9.87)	4.53 (2.71,7.59)	<0.001*
Wealth quintile	Lowest	9	97	1	1	
	Second	5	166	0.32 (0.10,0.99)	0.49 (0.14,1.67)	0.260
	Middle	19	116	1.76 (0.76,4.08)	1.35 (0.52,3.46)	0.532
	Fourth	24	117	2.21 (0.98,4.98)	1.41 (0.55,3.62)	0.468
	Highest	39	111	3.78 (1.74,8.21)	2.03 (0.81,5.08)	0.127
Accessibility of HF	<=5 km	86	404	4.32 (2.19,8.49)	3.17 (1.45,6.95)	0.004*
	>5	10	203	1	1	
ANC follow-up	No	15	204	1	1	
	Yes	81	403	2.73 (5.53,4.86)	0.46 (0.18,1.15)	0.059
Maternal education	No formal education	26	412	1	1	
	Primary	50	151	5.24 (3.15,8.73)	2.17 (1.05,4.49)	0.036*
	Secondary and above	20	44	7.20 (3.72,13.94)	1.53 (0.56,4.17)	0.397

## Discussion

This study mainly assessed the utilization of growth monitoring and promotion services among mothers with children aged 0–23 months in West Armachiho district, as these services highly affect the growth and development of the child in the early stages of life, reducing the risk of infections, morbidity, and mortality and decreasing mental, cognitive, and economic development. Accordingly, it was found that the utilization of growth monitoring and promotion services was 13.7%. This result is slightly higher than the study conducted in Butajira town, southern Ethiopia, which reported the utilization of GMP services to be 11% (22). This may be due to the involvement of urban kebeles together with the relatively low number of (four) rural kebeles in our study compared to the study conducted in Butajira, which included a high number of (nine) rural kebeles with no urban kebeles, which has an effect on their utilization of health services (23, 24), which in turn affects their exposure to GMP services (14, 17).

The result of this study is lower than the study conducted in different parts of Ethiopia, which reported the utilization of GMP services to be 16.9, 25.2, 32.9, and 38.9% (12–15). This difference may be due to a huge difference in place of delivery (as the health facility delivery for the last child in Banja district was 96.6%, while it was only 46.1% in our study) (12), which enables the mothers to get exposed to more information about the GMP and its advantages. Moreover, the difference in the number of respondents who were included in our study and the study conducted in Mareka district may be another reason for the discrepancy (13). However, a huge difference in knowledge and attitude about GMP services may be the source of the discrepancy in the proportion of GMP services (e.g., the proportion of good knowledge and favorable attitude about the utilization of GMP services was 54.6 and 56.2%, respectively, in a study conducted in Muhir Aklil district, while it was 19.3 and 8.8%, respectively, in our study).

In addition, the findings of this study are much lower than those of the studies conducted in Nepal, Northern Ghana, and Kenya, where the utilization of growth monitoring and promotion services was 90, 28.5, and 53.3%, respectively (25–27). This could be due to the difference in respondents’ backgrounds, as the respondents included in the study of Nepal were health worker mothers with child pairs and community health volunteer mothers with child pairs (25), while in this study, all mothers with children aged 0–23 months in the community were considered without any specification. Furthermore, the age of the children who participated in the study was younger than 5 years in the case of the study conducted in Northern Ghana (27) and 10–59 months in the case of the study conducted in Kenya (26).

In this study, delivery place, accessibility of health facilities, family size, knowledge of mothers/caregivers, and maternal education showed significant associations with the utilization of GMP services.

In this study, higher odds of utilization of growth monitoring and promotion services were observed among knowledgeable mothers/caregivers compared to their counterparts. This finding is supported by different studies conducted in Ethiopia, Ghana, and Bangladesh (16, 28–30). The reason could be that knowledgeable mothers become familiar with the importance of GMP in improving child health by understanding the information displayed on the growth chart, and preventive mechanisms of child malnutrition such as the timely initiation of breast milk, timely start of complementary feeding after the children reach the 6th month age, and continued breast feeding up to 2 years, which motivates them to use the services for their children.

Moreover, respondents who gave birth at a health institution were 3.16 times more likely to use growth monitoring and promotion services than those who gave birth at home. This result is supported by a study conducted in Ethiopia, Mareka, and Dabat districts (13, 31). This could be due to the fact that mothers who gave birth at a health facility have a higher opportunity to obtain nutritional counseling and counseling on child growth and development than those who gave birth at home.

In the present study, respondents who could access a health facility within 5 km or fewer had higher odds of using growth monitoring and promotion services than those who could not. This finding is strongly supported by the studies conducted elsewhere (30, 32, 33). This may be due to the fact that if the health facility is far away from their home, the respondents may not be able to access the health facility easily, which may prevent them from taking their children for growth monitoring and promotion services.

This study clearly presented that those respondents who have a family size of less than four had higher odds of using child growth monitoring and promotion services than those who have family members greater than or equal to four. In this study, it was found that the utilization of GMP services among respondents with a family size of less than four was 22.2%, while it was 11.6% among those who had a family size greater than or equal to four. The notion of our study is supported by a study conducted in Ethiopia (13). The reason for this may be that mothers who have low family sizes may have a minimum household workload, which helps them use different maternal and child health services, including GMP services, easily compared to mothers who have a high family size.

In this study, respondents who attended primary school were more likely to utilize growth monitoring and promotion services compared to those who did not have formal education. This result is supported by a study conducted in Ghana and Uganda (32, 34). This may be due to the fact that educated mothers/caregivers have a high level of health awareness and exposure to health information, which plays an important role in improving their health-seeking behavior so that they can utilize the GMP services in a well manner.

### Limitations of the study

Even though several actions (giving time to remember things that happened in the past, interviewing them alone where no one is around, etc.) were taken to minimize it, this study will not be free from recall bias and social desirability bias. Additionally, due to time and resource constraints, the study design applied to conduct this research is only quantitative. Moreover, there is a need for further qualitative studies regarding the utilization of GMP services.

### Conclusion and recommendation

The utilization of growth monitoring and promotion services in this study area was low. Maternal educational status, family size, accessibility of health facilities, delivery place, and knowledge of mothers/caregivers toward GMP services were found to influence the odds of using growth monitoring and promotion services. Therefore, giving due attention to the improvement of the knowledge of the mothers/caregivers about child GMP services and their advantages through nutrition education, together with counseling them to attend school, is recommended. Furthermore, it would be good if health facilities are built within a short distance of the community so that mothers/caregivers could easily access it to use the services. Moreover, providing health education to the mothers regarding health facility delivery and encouraging them to give birth at a health facility is better to enhance the utilization of child GMP services. Additionally, it is better to counsel the mothers to use family planning so that they may have a small family size, which helps them to use the services appropriately.

## Data availability statement

The raw data supporting the conclusions of this article will be made available by the authors, without undue reservation.

## Ethics statement

The studies involving humans were approved by Institutional Review Board, University of Gondar. The studies were conducted in accordance with the local legislation and institutional requirements. Written informed consent for participation in this study was provided by the participants’ legal guardians/next of kin.

## Author contributions

NT wrote the proposal, was involved in the study design, analyzed the data, and drafted the study. AA, BM, LB, and MS approved the design and proposal, and also involved in the data analysis, revised subsequent drafts of the study, and reviewed the manuscript. All authors read and approved the final manuscript.
